# Nurses’ perceptions of patient involvement in shared decision-making in cardiovascular care

**DOI:** 10.1016/j.heliyon.2023.e22890

**Published:** 2023-12-02

**Authors:** Eleni Siouta, Ulf Olsson, Nana Waldréus

**Affiliations:** a Sophiahemmet University, Stockholm, Sweden; b University of Stockholm, Stockholm, Sweden; c Department of Neurobiology, Care Sciences and Society, Division of Nursing, Karolinska Institutet, Stockholm, Sweden; d Theme Inflammation and Aging, Karolinska University Hospital, Huddinge, Sweden

**Keywords:** Atrial fibrillation treatment, Healthcare professional, Nurses, Shared decision-making

## Abstract

It is important for nurses to involve patients in their own care to enable shared decision-making. This study aimed to explore the perceptions of nurses regarding the degree to which involvement in shared decision-making takes place in clinical settings during consultations. Previous studies have shown that the use of shared decision-making by healthcare professionals can improve their caring practices and the quality of life of their patients. However, studies have also shown little evidence of the existence of shared decision-making in clinical practice. One step forward can be to clarify nurses’ perceptions of patient involvement in shared decision-making.

Qualitative data were collected from 10 nurses at four Swedish hospitals using a semi-structured, open-ended interview guide. The data were analyzed using inductive latent content analysis. The results showed that the care practices described by the nurses in the study are clearly different from the healthcare policy and scientific vision of shared decision-making. The nurses in the study believe that, with some exceptions, both healthcare professionals and patients prefer to leave decision-making to medical experts. In order to take advantage of the existing potential for improvement of shared decision-making in cardiologic care, healthcare professionals must be given time to seriously listen to and develop an interest in their patients’ lifeworlds. Furthermore, the implementation of shared decision-making requires a mutual initiative and development of knowledge about the decision-making process from healthcare professionals and patients.

## Summary statement

What is already known about this topic?

• Shared decision-making in healthcare is considered the core of person-centered care.
• The use of shared decision-making by healthcare professionals can improve their caring practices and the quality of life of their patients.

What does this paper add?

• It is important for nurses to involve patients in their own care to enable shared decision-making.
• The care practices described by the nurses in the study are clearly different from the healthcare policy and scientific vision of person-centered care.
• Nurses believe that both healthcare professionals and patients prefer to leave decision-making to medical experts.

What are the implications of this paper?

• In order to take advantage of the existing potential for improvement of shared decision-making in cardiologic care, healthcare professionals must be given time to seriously listen to and develop an interest in their patients' lifeworlds.
• The implementation of shared decision-making requires a mutual initiative and development of knowledge about the decision-making process from healthcare professionals and patients.
• According to the nurses, there is potential for improvement of involvement in shared decision-making.

## 1 Introduction

Shared decision-making in healthcare is considered the core of person-centered care and is defined as ‘the interpersonal, interdependent process in which health professionals, patients and their caregivers relate to and influence each other as they collaborate in making decisions about a patient's health [1,2].

Shared decision-making has to do with decisions that require attention through a collaborative process. Elwyn has argued that decisions must be sensitive to patient preferences which are most relevant and used where more than one alternative course of action is reasonable [3]. Shared decision-making involves both communication and collaboration, the ultimate meeting between experts, the patient and the healthcare staff [3]. This work requires the building of relationships, however short-term they may be, because shared decision-making requires all relevant participants to communicate openly and honestly. Sometimes it is the patient who expresses their goals, concerns, fears and preferences. In Siouta et al. [4], cardiologists stated that, by taking into account patients' feelings in the consultation and actively encouraging the patients to be involved, the cardiologists contributed to patient involvement in shared decision-making. The process can be called shared decision-making when the actors actively talk to each other, listen to each other and cooperate to arrive at a decision, even if the decision is to accept the current state or postpone action until another time [3]. Shared decision-making is considered important for improving the quality of cardiovascular care [5]. Patients with cardiovascular disease should be invited to be involved in making decisions relating to the appropriate treatment measures for their health problems and in implementing ways to manage their disease [6]. Previous studies have shown that interventions for increasing healthcare professionals' use of shared decision-making can improve their mental health-related quality of life but have provided little evidence of the existence of shared decision-making in clinical practice [1,3]. There may be a long way to go before shared decision-making becomes a constant part of clinical practice, but one step forward can be to clarify nurses’ perceptions of patient involvement in decision-making.

Involvement of patients in their own care is beneficial [3]. Patients with AF are confronted with choices of different treatments of AF that focus on reducing symptoms and preventing complications. Examples of such choices include medication, cardioversion and ablation techniques which are used to treat episodes of AF and maintain sinus rhythm, and these therapies often improve symptoms [6]. There is a difference between the decisions that are made in nursing and medical decision-making, i.e. at cardiology clinics, nurses make treatment decisions based on delegated responsibility within the medical knowledge domain. This responsibility is based on established guidelines and directives from cardiologists. However, examples of activities involved in the nurses' decision-making process in consultations include observations of signs that convey information on the patient's situation, confirmation of information gathered, and implementation of action strategies [7]. To achieve these efforts, the actual role of nurses during consultations should provide the patient with the necessary information so that the patient understands the purpose of the care and the nursing measures [8]. There is a significant responsibility to actively build relationships, referring to the person-centered approaches in care [9]. This part of nursing care contains aspects of interaction and communication. However, the results in Siouta et al. [10] indicate that patients seem to be more talkative and more likely to take an active role during nurse consultations compared to their encounters with physicians. The talk between the patients and physicians was more medically oriented as the physicians used ‘the medical voice’ to understand the patient's “experienced problems”. By talking from a more experience-based perspective, patients might integrate their knowledge with the medical-driven agenda; by listening to patients' stories, healthcare professionals might improve their knowledge, not only concerning the AF patient as a case, but also with regard to a patient's reactions to AF and how it has affected them personally [10].

In cardiovascular care, atrial fibrillation (AF) is the most common cardiac arrhythmia and is associated with poor health-related quality of life, increased hospitalization, morbidity and mortality [11]. About 37 million people worldwide suffer from AF [11,12]. AF means that the heart's normal impulse formation is affected by disturbances; thus, an irregular and rapid pulse occurs. This can lead to the heart's impaired pumping function, and thus the presence of stagnant blood in the heart's atrium, which can result in clotting [13].

The guidelines used when treating AF consist of acute rate and rhythm control (hemodynamic stability), management of precipitating factors (cardiovascular risk reduction, such as through lifestyle changes) and assessment of stroke risk (stroke prevention, such as oral anticoagulation), heart rate (symptom improvement and preservation of left ventricular function; rate control therapy) and symptoms (symptom improvement, such as antiarrhythmic drugs) [14]. Self-care is an essential part of the overall treatment, i.e. patients with AF must learn to manage their treatment and symptoms. Adoption of self-care activities can reduce the incidence of stroke, venous thromboembolism, heart failure, dementia and chronic kidney disease [15]. Unfortunately, education in supporting and encouraging patients with AF in their self-care is often lacking, and new ways to engage patients in self-care activities need to be explored [4,16]. Decisions regarding planning and execution of nursing interventions are planned based on the nurse's overall picture of the patient's resources and lifestyle. It is the nurse's responsibility to review knowledge gaps and educate and motivate patients to reduce the risks of suffering from AF [17]. It is therefore important that the nurse promotes patient participation and clarifies the patient's position in a treatment decision.

One thread of argument in the literature on patient engagement in shared decision-making is that shared decision-making in healthcare is crucial because patients must be involved in their own care and want to have a more active role in it [1]. Shared decision-making can help clarify alternatives in care when advantages and disadvantages are discussed, reduce unjustified care practice variations, and promote healthcare sustainability by increasing patients' ownership of their own healthcare [1]. Involving patients in their own care is essential for many reasons, such as allowing their beliefs, values and preferences to be incorporated into their healthcare and into the shared decision-making processes [18,19]. Patients' involvement and participation in their own care is an effective and empowering healthcare tool that can be made part of patients' self-management [20]. Obtaining clarifications about AF and its treatment, cooperating in decision-making with healthcare professionals, and acquiring knowledge and feeling understood can enhance patients’ involvement and help them make autonomous decisions [4]. Furthermore, if patients are involved in the decision-making regarding their own treatment, they tend to comply with the treatment, which in turn leads to better outcomes [21].

Several intervention studies for increasing healthcare professionals' use of shared decision-making have been published (87 studies in 2017 alone) [1]. Fifteen studies were in the field of cardiovascular care, and most of the interventions (n = 44) targeted patients. Only 15 interventions targeted healthcare professionals. Overall, the effectiveness of the interventions is unclear because of scant evidence for such [1]. Some more recent studies have described how shared decision-making interventions are implemented and routinized in clinics and, more relevantly, the comparative effectiveness of these implementations has also been studied [6,22]. It has been observed that patient involvement can conflict with cardiologists' other concerns and values with regard to their professional responsibilities [[23], [24], [25]]. If cardiologists viewed patients with AF as active participants and collaborators in their own healthcare processes, then it would be possible for them to involve their patients in shared decision-making [25]. In addition, patient involvement strategies could be developed in subsequent communications and interactions [7]. Previous studies have explored shared decision-making from the perspective of patients [4,25]. Despite not being actively involved in shared decision-making, the patients can feel involved through experiencing supportive communication. In consultations with nurses, the patients can feel involved when they receive clarification and when preparing for and building up confidence in decision-making. Less is known about nurses' views on patients' experiences of shared decision-making and how they involve their patients in it. Therefore, this study aimed to explore the perceptions of nurses regarding the degree to which involvement in shared decision-making takes place in clinical settings during consultations about atrial fibrillation treatment. However, only nurses are interviewed in the study, i.e., not doctors and patients. This means that the study is focused on nurses' subjective perceptions of nurses', doctors' and patients’ approaches to shared decision-making.

## 2 Method

### 2.1 Design and setting

This study was based on a descriptive qualitative design, meaning that epistemologically and ontologically the study's results are based on the researcher's interpretation of the informants' subjective perceptions [26]. This means that the study is focused on nurses' subjective perceptions of nurses', doctors' and patients' approaches to shared decision-making. The data analysis is inductive, which means that the categories that structure the presentation of the study's results have emerged from the empirical material [26]. The interviews were conducted at four general cardiology clinics in Sweden from January to December 2015. The cardiology clinics were strategically selected based on localization and size, focusing on university and county hospitals, thus enabling description of different views [26]. The research team consisted of associate professors ES and UO, and assistant professor NW, all of whom have previous experience of working with qualitative studies.

### 2.2 Participants and recruitment

A purposeful sampling method was used, and the inclusion criterion for participants was nurses working at a cardiology clinic with patients with AF. There were no exclusion criteria. Potential participants were contacted via e-mail by the first author, with information about the study and the study's purpose. Ten participants who were interested were scheduled for a 1-h appointment for the interview. No relationship was established with the participants prior to the interviews, and the participants had no personal information about the interviewer. None of the participants dropped out. The participants' characteristics are shown in Table 1.

**Table 1:** Characteristics of the nurse participants (n = 10).

Variable	Nurses n = 10
Gender	
Male/female	7/3
**Age,** range years	30–57
Type of hospital	
University	7
County	3
**Clinical experience in cardiology,** years	
Fewer than 2 years	1
2–10 years	5
11–20 years	4

### 2.3 Data collection

Interviews were conducted by the first author (ES) at the participants' offices in their workplaces. Each interview lasted for 20–40 min and was audio-recorded. A semi-structured interview guide was developed based on topics that were relevant to patient involvement in shared decision-making as inspired by the OPTION instrument [27]. The OPTION instrument consists of 12 items, divided into five core dimensions [1]: Constructive interpersonal engagement [2], recognition of alternative actions [3], comparative learning [4], preference construction and elicitation, and [5] preference integration. Informants were asked to reflect on each item, including factors influencing patient involvement in the decision about AF treatment. Important aspects of patient involvement in shared decision-making regarding AF treatment were considered, with questions such as ‘How do you [the nurses] involve patients in the decision-making regarding treatment in the consultations?’ and ‘What preconditions facilitate patient involvement in the decision-making regarding treatment?’ The first interview was conducted as a test interview to improve the interview guide and questions.

When interviewing, the researcher reflected on the initial information and considered whether there are further questions that need to be asked to gain a deeper understanding of the topic [26]. The researcher identified no gaps in the collected material and concluded the recruitment process when sufficient saturation was achieved after ten interviews.

### 2.4 Data analysis

The analysis of data was based on a descriptive qualitative design and thus the researcher's interpretation of the informants' subjective perceptions regarding the degree to which involvement in shared decision-making takes place in clinical settings during consultations.

The data analysis was inductive, which means that the categories that structure the presentation of the study's results have emerged from the empirical material [28]. We distinguish between the informants' statements and the authors' interpretations of the statements.

The researchers used content analysis with qualitative method, and in an inductive manner [28]. Creating categories is characteristic of qualitative content analysis. A category is a group of content that shares a commonality; a category answers the question ‘What?’ and can be identified as a line throughout the codes [28]. Important concepts related to qualitative content analysis include manifest and latent content, unit of analysis, meaning unit, condensation, abstraction, code, category and theme. In research using qualitative content analysis, a systematic procedure is used to extract the main content [28]. The analysis was conducted by two authors (ES and UO) and consisted of the following steps: Initially, verbatim transcripts of all interviews with the nurses were produced. The interview transcripts were read multiple times in search of statements describing experiences related to patient involvement in shared decision-making regarding AF treatment. The sentences that had the same meaning were condensed and coded by the first author. Similar codes were grouped together and, by comparing differences and similarities, subcategories and main categories were created. The codes were then transferred to a coding page, where similar codes were grouped together. By comparing the codes to determine the differences and similarities between their content, subcategories and main categories were created. To gain an overall impression, the researchers (ES and UO) placed the condensed meaning units that showed similarities into new, coded groups related to the aim of the study. The categories were carefully reflected by all three authors upon nurses' experiences related to patient involvement in shared decision-making and discussed in repeated analyses until consensus was achieved.

### 2.5 Ethics

The study was performed in accordance with the International Code of Medical Ethics adopted by the World Medical Association [29,30]. Although the Declaration is addressed primarily to physicians, the WMA encourages other participants in medical research involving human subjects to adopt these principles. All participants provided informed consent before data collection, and they were assigned a code to guarantee confidentiality and anonymity. Ethical approval was obtained from the Regional Ethics Committee in Linköping, Sweden (Ref. 2014/146–32).

## 3 Results

Three categories were derived from the interview data. The first category describes *the nurses' perceptions of patients' desire to participate in shared decision-making.* The second category concerns the nurses' perceptions of the decision-making process between the parties. Finally, the third category describes the nurses' perceptions of the crucial factors for patients' active involvement in decision-making. The results are summarized in Table 2. Appropriate interview transcript excerpts showing the nurses’ perceptions are presented to illustrate the categories.

**Table 2:** Findings presented as categories and subcategories.

Categories	Subcategories
***Nurses' perceptions of patients' desire to participate in shared decision-making***	Patients follow professionals' decisions Decision-making authority in the acute disease stage
**Nurses' perceptions of the decision-making process between the parties.**	The doctor recommends and the patient takes a stand
	Persuasion to get the patient to change their mind
	Hanging on when the patients take the initiative
	Responding to the patient's concerns and fears
**Nurses' perceptions of crucial factors for patients' active involvement**	The patient's age and education
	The patient's level of knowledge and need for information
	Environment and time as prerequisites for conversation
	The nature and severity of the decision

### 3.1 Nurses' perceptions of patients’ desire to participate in shared decision-making

#### 3.1.1 Patients follow professionals’ decisions

According to the nurses, patients usually follow the professionals’ decisions; few speak up and refuse to do so.

*Most of the time, it feels like the patients leave themselves in our hands.… They are very grateful for the medical examinations we offer.* (Nurse 4)

According to Nurse 4, patients do not want to participate in the decision-making regarding their own treatment. However, the same nurse also described an incident in which the patient refused to do what the doctor had previously decided.

*For instance, last week, a patient told me, ‘I don’t want to [follow the doctor’s decision].’ I spoke to the doctor; he went in and the patient got what he wanted.* (Nurse 4)

As the nurse in the aforementioned case did not have the mandate to make the type of decision called for, she turned to the doctor, who altered his previous decision and agreed to the patient's request.

#### 3.1.2 Decision-making authority in the acute disease stage

Especially in emergency situations, patients want someone else to take responsibility and decide what to do because they feel safe with that.

*Patients in the acute disease stage often feel very safe if I’m paternalistic with them. ‘This is what we plan to do’, I tell them, and they feel that I can do something and know what to do. This makes them relax. They consider it nice that someone takes care of the matter and does something.* (Nurse 3)

According to nurses, the patients often leave the decision-making to the professional because they trust the latter's medical expertise and feel safe with them.

### 3.2 Nurses’ perceptions of the decision-making process between the parties

#### 3.2.1 The doctor recommends and the patient takes a stand

Usually, the doctor recommends a certain treatment or medication, and the patient decides on the matter by saying ‘yes’ or ‘no’.

*Yes, I think it’s better that you recommend, that the patient is not part of the decision-making process. You simply say, ‘This is what we would recommend to be done’ or ‘This is the next step that we must take’.* (Nurse 3)

Thus, patients are not particularly actively involved in the decision-making processes. Most of the time, they just accept the doctors' suggestions. However, occasionally, the patient declines to follow the doctor's suggestion. Nonetheless, nurses never try to force patients to accept what has been recommended.

*We don’t force treatment on anyone. If they don’t want it, it’s their decision in the end.* (Nurse 2)

As it is neither possible nor desirable to force patients to accept the doctors' recommendations, other strategies must be chosen to get patients to accept these.

#### 3.2.2 Persuasion to get the patient to change their mind

If the patient says ‘no’ to the doctor's recommended treatment, the doctor often tries to persuade the patient to accept the recommendation by informing them about the advantages and disadvantages of the recommended treatment, such as by citing the latest relevant findings.

*But then there’s no participation [on the part of the patient]. The doctor tries to persuade the patient, for their own good …. The doctor has good paternalism, so to speak. Yes, they want what’s best for the patient.* (Nurse 5)

According to Nurse 5, paternalism in the form of persuasion is not always bad; it can also be good if it is for the patient's own good. For example, it may involve patients who have had fibrillation for a long time but are worried about his-ablation and do not understand that they will feel much better after undergoing the treatment. However, according to Nurse 5, it seems that patients do not always realize what is in their best interests. In these situations, both the doctor and the nurse have to ‘coax’ the patient. This can be done, for example, for patients who want to stay in bed day after day.

*I try to coax [the patient] as best I can to make them understand that what they want is not the best for them, but it happens that I make the decision and say, ‘You have to do this, for your own good’. I then almost force the patient to get up. Afterwards, you get a reply like ‘Thank you very much!’* (Nurse 6)

According to Nurse 6, healthcare professionals' attempts at persuasion and coaxing sometimes become almost coercive as this is their last resort for getting the patient to accept what the doctor deems best for them. Nurse 5 said that they sometimes have to ‘almost persuade the patient in an ugly way’, such as shifting the responsibility for what may happen to the patient if they continue to reject the doctor's recommendation.

*We sometimes tell the patient, ‘We recommend this, but if you don’t want it, then it’s up to you. Whatever happens, it’s your responsibility.* (Nurse 2)

Often, the aforementioned statement puts pressure on the patient, ultimately leading them to accept the doctor's recommendation.

#### 3.2.3 Hanging on when patients take the initiative

Sometimes, the question of alternative treatments arises. However, according to Nurse 2, it is usually not the doctor or the nurse but rather the patient who brings up and wants to discuss alternative forms of treatment.

*They ask, ‘Why not this?’ or ‘Why that?’ They also ask about pros and cons, about the latest findings, and so on, so you can have that discussion with the patient.…* (Nurse 2)

According to Nurse 2, when patients take the initiative and ask questions about different treatment options, healthcare professionals perceive that they are ready to take on different points of view and discuss the advantages and disadvantages of different treatment options.

*Most of the time, we tell the patients about the pros and cons of the alternative treatments, and then we notice that they are ready to take these in.* (Nurse 3)

Nurse 3 believes that it is good when the patient asks the aforementioned types of questions because this indicates that it makes sense for the doctor to invest time in discussing the different treatment options with the patient as the latter is ready for it. If the patient is not ready for it, according to several nurses, it is risky to bring up different treatment options, as the patient's concern may be diluted.

#### 3.2.4 Responding to the patient's concerns and fears

Most patients are worried about both their disease and the treatment that they will most likely have to undergo.

… *In addition to Waran, there’s also ablation. Most patients are afraid of this. They usually call at the last minute and say that they don’t want to undergo ablation because they think it’s scary to have a catheter inserted into their groin*. (Nurse 4)

Patients are worried about both the medication they will be given and other interventions they may receive, such as ablation. This is often because of the information about these that they obtain from mass media and from their friends and acquaintances who have previously received such interventions.

*Waran is often associated with problems. Many patients read from newspapers that you can die from the treatment, that it’s rat poison, and so on.* (Nurse 9)

When patients’ anxiety becomes too strong, they wonder if the recommended treatment will really be good for them. This can sometimes lead them to make ill-considered decisions. Then, the nurse must again discuss with them the advantages and disadvantages of the treatment and the risks of stopping the medication. The anxiety is usually greater in new patients; those who have been around longer are often calmer, although more experienced patients can also be anxious.

*If they come in for the 10th time, they are somehow calm because they have been part of the process before.* (Nurse 3)

Thus, even patients who have received the same treatment many times can have concerns regarding it, often about how many times they can undergo the treatment.

*Well, they’re here again. Their concerns are annoying, but I confirm these*. (Nurse 3)

Patients’ concerns do not disappear even if they have received the same treatment or intervention before and may already have sufficient information and knowledge about it. Even experienced patients feel anxiety and need confirmation of this.

### 3.3 Nurses' perceptions of crucial factors for patients’ active involvement

#### 3.3.1 The patient's age and education

As can be seen in the aforementioned quotes, most patients prefer to leave the decisions that must be made to the doctor and nurse. This is especially true for elderly patients. They often view the doctor as an exalted person who is all-knowing and powerful and who can and must simply be trusted. Younger patients, on the other hand, who often have more education, want to be more involved in their own care and often ask various questions concerning the entire care process.

… *while the 40-somethings and the younger ones who come here are better educated. They have googled the treatment options and gathered knowledge about them. They also have their own ideas, which make them question a little more and want to be more involved in the process.* (Nurse 3)

The younger patients who come to the hospital more prepared have often acquired relevant knowledge through sources other than the treating doctor. Thus, they can ask questions, have their own ideas, and question what the doctor says. Sometimes they have even ‘diagnosed themselves’ (Nurse 10).

Furthermore, the nurse participants believe that some differences can be seen between male and female patients. Female patients usually ask more questions and call more often than male patients do. This pattern is particularly true for women with other cultural backgrounds.

*For some patients who come from other cultures, having their loved ones here is a matter of course. So, you also have to inform them of the treatment options, and they also have a lot of questions all the time.* (Nurse 3)

According to Nurse 3, it seems more natural for non-Swedish patients to involve their relatives in their care. However, even if the interest in actively participating in one's own care can differ between different patient groups, this difference should not be exaggerated, according to several nurses.

*Well, I think it depends on the individual.… I don’t want to generalize in any way.* (Nurse 1)

According to several nurses, they cannot generalize when it comes to non-Swedish patients involving their relatives in their care because it can vary from person to person as each patient is unique and therefore approaches matters in different ways.

#### 3.3.2 The patient's level of knowledge and need for information

As we have already seen, some patients manage to acquire information about treatment options on their own. However, other patients do not understand anything about the treatment options and find it difficult to take in the information provided by brochures or what is brought up in the conversations that occur during the doctors’ visits.

*… sometimes you think that the patient and you have already completely agreed on something, but in the next meeting, you realize that it wasn’t like that at all. Then, you have to start all over again. You have to re-evaluate the patient and try to find out where they are.* (Nurse 1)

*I see right away that it doesn’t matter what I say now because they’re not there. I have to limit the information I will give them and must consider where they are, or it will all be lost. If I can get even just some information into their heads, maybe they can absorb it. The more information they take in, the more it gets lost.* (Nurse 2)

Therefore, in the nurse's meeting with the patient, it is important for the nurse to grasp where the patient is in terms of knowledge and what they have come to understand. The nurse should start from where the patient is and rehearse, explain and provide additional information to them.

Thus, for some patients, especially those who are anxious, it may be important for the nurse not to provide all the necessary information at the same time because anxiety may serve as a barrier to assimilating information. Several nurses emphasized the importance of supplementing their conversations with patients with written information that the patients could take home and read in peace and quiet.

*They always call and ask for advice. This happens all the time – patients asking for advice, what to think about, if there’s something in the brochure that they don’t understand.* (Nurse 4)

Most patients appreciate receiving written information about the treatment options because there is so much for them to take in during the calls. However, this does not always help; sometimes, they come back after a few weeks and still do not understand.

#### 3.3.3 Environment and time as prerequisites for conversation

According to the nurse participants, the external conditions of nurses’ conversations with patients are important. It is best if the nurse sits alone with the patient and converses with them.

*But usually and unfortunately, our room is divided into four parts, so it’s hard to sit alone with the patient. You often converse with the patient when there are other patients around*. (Nurse 4)

As can be seen in the above quote, most of the time, it is not possible for a nurse to sit in a private room with a patient. Lack of time also limits nurses’ opportunities to seriously listen to patients and involve them in the care process. This is largely due to a large number of patients and the lack of hospital staff.

*That’s what it’s all about, so the National Board of Health and Welfare can write what they want if we don’t get time with the patient … We are on minimum staffing here and work overtime. Time matters, and there’s no time to sit …. Yes, I can’t sit there for 20 minutes because there are 10 other things I have to take care of as I’m alone. So many other things must be done, and there are alarms. I don’t have time for that.* (Nurse 8)

Thus, according to Nurse 8, it does not matter what the healthcare authorities strive for; if the hospital staff are not given the temporal conditions needed to realize it, it will not be attained. However, the conditions seem to be deteriorating because the tendency is the opposite.

*That’s probably what we’re getting more and more of. There’s less and less staff, and there’s more and more lack of time.* (Nurse 10)

As a consequence of the aforementioned, both doctors and nurses have to find ways to speed up their conversations with their patients to *save* time, such as by not answering some of their questions.

*It’s very simple and time-saving to say to a patient, ‘You have this problem. We can do this to treat you’. Then, the patient doesn’t have to make the decision themselves … and we won’t need to explain what it would be like for them to live with their chronic ailment and ask how they are really doing, so you save a lot of time.* (Nurse 9)

When you know that the patient waiting list is long, the easiest and most time-saving thing to do is to try to speed up the call. In other words, there is much administrative work that takes soul time from the patients who are waiting.

*Time is a problem due to too much other shit, sorry …. Yes, quality register and senior alert and all these. These take our time away from the patients*. (Nurse 8)

However, in a situation where there is a constant lack of time, it becomes particularly important to at least give the patients time at the first visit because ‘then, you notice that there are fewer questions the week after’ (Nurse 6).

#### 3.3.4 The nature and severity of the decision

One of the nurses wondered what shared decision-making meant, especially when it came to problems like AF and medication for it.

*If the heart slows down, it can cause complications that are worse than walking with fibrillation. Participating in decisions is, well, I don’t know … what could it be?* (Nurse 8)

Even if Nurse 8 is hesitant to involve the patient in matters such as the choice of medication, she believes that it may be possible to involve the patient in making other types of decisions.

*Yes, maybe to go to the X-ray room eventually …. I say, ‘Do you want to join us? We will take some blood samples because of this and this’. Roughly that. But in emergency interventions – that’s what they’re called – you can’t just stand there and shillyshally.* (Nurse 8)

Nurse 8's use of the phrase ‘just stand there and shillyshally’ can indicate complete opposition to involving the patient in decisions that could have serious consequences.

## 4 Discussion

The most important finding related to the study's aim of exploring nurses' perceptions of patient involvement in shared decision-making was that the practice described by the nurse participants could not be considered person-centered. Patients usually do not participate seriously in shared decision-making and do not appear as participants in a care team consisting of healthcare professionals and patients and as active resources in their own care. This is perhaps not surprising because, as perceived by the nurse participants, neither the doctors nor the nurses seem to have a particularly strong desire to seriously involve the patients in their own care and in shared decision-making, and the patients do not show a desire to participate in these. In the nurses' descriptions of concrete nursing practice, the patient's general tendency is to leave themselves in the hands of the medical experts. To some extent, this contradicts Légaré et al.‘s [1] claim that patients generally want to play a more active role in their own healthcare. However, when it comes to younger patients and women, the results of the study correspond to some extent with Légaré et al.‘s claim; that is, younger patients more often want to be actively involved in their own healthcare. However, generally, according to the nurse, patients prefer to leave the decision-making to the healthcare professionals, which doctors and nurses do not actively counteract because they prefer to be the ones controlling the care process, which was also found in other studies [[23], [24], [25]]. The results of the present study thus indicate that the parties involved in healthcare have, more or less, a mutual interest in preserving traditional power relationships. The mutual desires and aspirations of the parties involved differ from the vision of involvement and shared decision-making constructed in both the health policy and health science contexts. Thus, as also Légaré et al.‘s [1] claim, in the meetings between healthcare professionals and patients, the included parts do not collaborate with each other in a way that demonstrates involvement and shared decision-making and supports the patient to make autonomous decisions. This means that the meetings between patients and healthcare professionals are not used as learning arenas to develop patients' self-management [31] and self-care activities [15,32]. Most often, both nurses and doctors use recommendations and persuasion, which, according to the nurse participants, sometimes makes the patients feel that they are being forced to accept the doctors' recommendations. However, the picture that emerges from the nurse participants' descriptions is not unambiguous. According to the nurses, doctors, nurses and younger patients sometimes demonstrate a desire to develop care in a way that will make the patients more involved. In healthcare practice, it is often the informed patients who ask questions about different treatment options in a way that makes the healthcare professionals hang on because they perceive such patients as being prepared to discuss different treatment options. Thus, there is potential for improvement in shared decision-making. The question then becomes ‘What conditions are required to utilize the potential for improvement in shared decision-making that, according to the nurse participants, exists?’ An important prerequisite is to create more time for patient contact in various ways, such as by enabling undisturbed physical meetings and phone calls. According to the nurse participants, the time that they can spend conversing with individual patients is currently severely limited due to a large number of patients, nurses' extensive administrative tasks, and lack of staff. Overall, these factors limit the possibilities for person-centered care as time is needed to verbally and non-verbally encourage patients to share their life stories, expectations and questions, and to listen to patients and alleviate their fears and anxieties. Another important factor in making patients become active participants in their own care and in the decision-making process is the level of the patient's information, education or knowledge. Patients can acquire the required knowledge on their own via the Internet or can be offered knowledge and information within the framework of the care process itself. This requires both that they actively seek knowledge and information and that nurses actively listen to patients, ask questions and offer knowledge and information to them. However, even if time is an important prerequisite, more time does not automatically make care more person-centered. A study by Siouta et al. [25] showed that even consultations in which time is not a limiting factor tend to be limited to the world of medicine, at the expense of the patients' lifeworld. Nurses need to find out what expectations of participation the patient has, and in what way they want to be involved in shared decision-making. It is the nurses that must create the conditions for patient participation, and it is the patient's definition and prioritization of aspects for participation that must be given priority.

### 4.1 Strengths and limitations

The credibility of the results is the foundation of high-quality qualitative research [33]. The criteria included strategies such as developing guidelines for data collection, defining and obtaining adequate participation, achieving data saturation and ensuring high levels of consistency and agreement between coders. One limitation is that member checking was not conducted. In the present study, data were collected from interviews with only 10 nurses. An adequate number of nurses have been selected to achieve values in the findings from the qualitative content analysis. The study had a descriptive qualitative research design, and a statistically representative sample was not obtained. The nurse participants' answers were also not quantitated, although the use of interviews with a clinical sample (Table 1) and qualitative content analysis identified new and relevant aspects of patient involvement in decision-making during medical consultations. Another limitation of the present study was that all 10 nurses who were interviewed were from a single country or culture. Thus, their biases or worldviews are likely to be similar. A potential limitation could be that the interviews were conducted in Swedish and translated into English, which may have influenced the interpretation of the nurse participants' statements. Finally, despite these study limitations, the nurses in the sample, men and women who had practiced cardiologic care for 2–20 years, were strategically selected to strengthen the study [26]. Therefore, we believe that the data provided by the study's findings have a high value.

### 4.2 Trustworthiness

In our qualitative interview study, we applied Guba and Lincoln's framework for credibility to enhance trustworthiness [34]. We provided comprehensive descriptions of the research context, participant profiles, and data collection methods. Reflexivity was diligently practiced to address personal biases and maintain objectivity. In qualitative research, researchers must be conscious of their own role in the research process (i.e. taking this into account and acknowledging their own perspective and voice) [26]. To control for bias, it is important that the researcher is aware of his or her own preconceived notions. We worked actively to reduce our own impact with insider perspectives when conducting research and interviewing nurses. This may include having an open mind when asking questions and listening to the nurses' answers without predicting or directing the answers. It is also important to be aware of any prior assumptions and not allow these to influence the data analysis [35]. Additionally, reflections with the research group forced us to explicitly express our interpretations and what we based our interpretations on. Reflexivity helped us, as researchers, to be objective and to analyze and interpret data in a nuanced way. In conclusion, upholding the trustworthiness of our qualitative inquiry necessitated vigilance in addressing personal biases, conscientious reflexivity and the application of rigorous validation mechanisms. These efforts collectively fortified the reliability and credibility of our research outcomes.

### 4.3 Implications for practice

There is potential for improvement in shared decision-making. The implementation of shared decision-making requires a mutual initiative and a mutual development of knowledge about the decision-making process from healthcare professionals and patients. To capitalize on the potential for improvement of shared decision-making in cardiac care, healthcare professionals must be given time to seriously listen to and develop an interest in their patients' lifeworlds. The nurses have a central role in supporting the patients’ decision-making process by finding out expectations about how they want to be involved in decision-making.

## 5 Conclusion

The study shows that the care practices described by the nurse participants are different from those in the healthcare policy and scientific vision. In the nurses' view, the patient's general tendency is to leave themselves in the hands of the medical experts. And the conditions required to seriously involve patients in shared decision-making are largely lacking, according to the nurses. This, above all, is due to the fact that, according to the nurses, healthcare professionals do not have the practical means (e.g. time) to seriously listen to each patient and familiarize themselves with the parts of the patients' lifeworlds that are relevant to the decision-making process. Thus, the parties involved in healthcare seem to have a mutual interest in preserving traditional power relations. However, the study also indicates that, according to the nurses, there is potential for improvement of involvement in shared decision-making.

## Ethics statement

Ethical approval was obtained from the Regional Ethics Committee in Linköping, Sweden (approval number 2014/146–32). The study complies with all regulations and all participants provided informed consent before study participation. The research conducted in this study adheres to the ethical guidelines and declarations as outlined in the updated ethics declarations list available at https://www.cell.com/heliyon/ethics.

## Funding statement

This research has no funding.

## Data availability statement

The authors do not have permission to share data. The data associated with our study has not been deposited into a publicly available repository.

## CRediT authorship contribution statement

**Eleni Siouta:** Writing – review & editing, Writing – original draft, Supervision, Project administration, Methodology, Investigation, Formal analysis, Data curation. **Ulf Olsson:** Writing – review & editing, Writing – original draft, Formal analysis. **Nana Waldréus:** Writing – review & editing, Writing – original draft, Conceptualization.

## Declaration of competing interest

The authors declare that they have no known competing financial interests or personal relationships that could have appeared to influence the work reported in this paper.
